# Making Sense of Self Talk

**DOI:** 10.1007/s13164-017-0375-y

**Published:** 2017-12-28

**Authors:** Bart Geurts

**Affiliations:** Philosophy Department, University of Nijmegen, Nijmegen, Netherlands

## Abstract

People talk not only to others but also to themselves. The self talk we engage in may be overt or covert, and is associated with a variety of higher mental functions, including reasoning, problem solving, planning and plan execution, attention, and motivation. When talking to herself, a speaker takes devices from her mother tongue, originally designed for interpersonal communication, and employs them to communicate with herself. But what could it even mean to communicate with oneself? To answer that question, we need a theory of communication that explains how the same linguistic devices may be used to communicate with others and oneself. On the received view, which defines communication as information exchange, self talk appears to be an anomaly, for it is hard to see the point of exchanging information with oneself. However, if communication is analysed as a way of negotiating commitments between speaker and hearer, then communication may be useful even when speaker and hearer coincide. Thus a commitment-based approach allows us to make sense of self talk as well as social talk.

## Introduction

We talk a lot. Much of our talk is addressed to others: this is the kind that the humanities and social sciences have lavished attention upon. But people talk to themselves as well, and this is a phenomenon that has received much less attention and is not nearly as well understood. Nonetheless, self talk has long been recognised as a central aspect of our mental lives. This view is firmly entrenched in the vernacular: wondering if it will rain tomorrow, I “ask myself” whether it will rain; I “tell myself” to do the dishes and “promise myself” an espresso when I’m done; I “remind myself” to lock the door when leaving my office; and so on. Asking, telling, promising, and reminding are originally social acts, but when addressed not to others but oneself, they come to function as modes of thinking: wondering, making up one’s mind, motivating oneself, and so on. My purpose in this paper is to understand how that can be; how social acts get transformed into private acts.

The connection between thinking and self talk was made already by Plato, ca 369 bc, who defined thinking as “the conversation which the soul holds with herself in considering of anything. […] The soul when thinking appears to me to be just talking — asking questions of herself and answering them, affirming and denying.” (*Theaetetus* 190a, translation by Jowett 1871) Since Plato, the idea that self talk is an essential part of human psychology has been endorsed by aformidable cast of philosophers and, more recently, psychologists. Amongst the latter, Vygotsky deserves special mention for highlighting the developmental aspect and canvassing the view that self talk develops out of, and is continuous with, social talk. According to Vygotsky, the development of self talk is part and parcel of the development of higher mental functions, all of which originate from the internalisation of social interaction:

Children master the social forms of behaviour and transfer these forms to themselves. […] Ishall relate to myself as people relate to me. […] Any function in the child’s cultural development appears twice, or on two planes. First it appears on the social plane, and then on the psychological plane. First it appears between people as an interpsychological category, and then within the child as an intrapsychological category. This is equally true with regard to voluntary attention, logical memory, the formation of concepts, and the development of volition. […] All higher mental functions are internalised social relationships. (Vygotsky 1981: 158, 163-164)

Although Vygotsky’s views may have been a bit extreme, it is uncontroversial that ours is a world of social interactions, especially communicative interactions, which shape our behaviour in all sorts of ways. Speech acts start their career as a form of social interaction, but almost as soon as they begin to talk, children will also talk to themselves, using speech acts to shape their own behaviour. For example, a mother tells her son not to touch the toaster, and later on, when the boy feels drawn towards the toaster, he restrains himself by means of his mother’s words: “Don’t touch the toaster!” Thus social talk becomes private talk, which at first is mostly overt, but is increasingly internalised to become inner speech, or “verbal thought”.

In order to account for the transition from social talk to self talk, we need a theory that equally covers other-directed and self-directed communication. The main objective of this paper is to provide such a theory. Given that the transition from social talk to self talk is such a smooth one, it is not enough to have plausible accounts of each: what is called for is a unified account that captures the continuity between the two. The received view on communication fails this requirement. According to that view, communication is information exchange, and generally speaking, information exchange presupposes that sender and receiver are distinct individuals. The problem is that, in self talk, sender and receiver seem to coincide. That’s why it feels unnatural to paraphrase the little boy’s self-addressed directive, “Don’t touch the toaster!”, as informing himself that he mustn’t touch the toaster.

Communication is first and foremost a social practice, whose basic units are speech acts. Self talk is a private practice that develops out of social talk, and therefore it makes sense that we should seek to capture the continuity between the two practices by way of a theory of speech acts. This is an important point, because speech act theories are not processing theories, and existing accounts of self talk are usually cast in terms of mental representations and processes. Hence, the general approach I advocate, though unsurprising from a pragmatic point of view, is quite different from existing accounts of self talk.^1^

The remainder of this paper is laid out as follows. To begin with, I survey the key facts about self talk and then proceed to discuss why the information-based view on communication fails to make sense of this phenomenon. Adopting an idea that has been common currency in speech act theory since Austin (1962), I then develop an alternative view, according to which communication is primarily a matter of negotiating commitments, and I argue that this approach equally applies to social talk and self talk.

## Self Talk

To a large extent, Vygotsky’s views on self talk have been corroborated by experimental data (see Winsler 2009 and Vicente and Martinez Manrique 2011 for surveys and references). Most normal-developing children begin to engage in self talk in their second or third year of life, around the time they start speaking in sentences. Initially, self talk is fully overt and not always clearly distinct from other-directed speech. Its use builds up until the fifth year, after which a slow process of internalisation sets in: self talk gradually becomes more truncated and harder for overhearers to follow, while more and more children report using inner speech.^2^ However, overt self talk never goes away entirely, and it remains in use throughout the lifespan.

Communicating with ourselves comes naturally to us. We don’t teach our children to talk to themselves, and even before they start speaking, children use a variety of gestures and points to share information with others, but will also gesture and point for themselves (Rodríguez and Palacios 2007; Delgado et al. 2009). Deaf children spontaneously sign for themselves just as hearing children talk to themselves (Kelman 2001; Gutierrez 2006). More remarkable still, self-address seems to come naturally to other species, as well: there is good evidence that non-human primates who have been taught the basics of a sign language are liable to spontaneously sign for themselves (Bodamer et al. 1994; Jensvold 2014). For example, Gardner and Gardner (1974) report that their foster chimpanzee Washoe was often seen “moving stealthily to a forbidden part of the yard signing ‘quiet’ to herself, or running pell-mell for the potty chair while signing ‘hurry’.” (p. 20)

All of us talk to ourselves at least some of the time, though we are not always aware of doing so (Duncan and Cheyne 2001; Winsler et al. 2006), and though proportions vary, many people spend a good deal of their waking lives engaged in self talk. In fact, it is quite possible that some of us spend more time talking to themselves than to others (Heavey and Hurlburt 2008). The purposes of self talk are likely to be as many and as varied as the purposes of “normal”, other-directed talk. Self talk has been shown to be associated with a variety of psychological functions, including reasoning, problem solving, planning and plan execution, attention, and motivation (Winsler 2009; Vicente and Martinez Manrique 2011). Self talk is triggered by all sorts of problem situations, including paper folding, taking exams, and entering data on a computer, as well as by problem complexity (Duncan and Cheyne^2001^, Duncan and Tarulli 2009). For example, in a study by Fernyhough and Fradley (2005), 5- and 6-year-old children were presented with puzzles of varying degrees of complexity, measured in terms of the minimum number of moves required to solve the puzzle. It was found, first, that levels of overt self talk were positively correlated with task performance, and secondly, that there was a ∩-shaped relation between levels of self talk and task complexity. Apparently, children engaged in self talk when the task got harder, and gave up when it became too hard.

Unlike overt self talk, covert self talk is not readily observable, and therefore harder to probe experimentally. Nevertheless, as attested by Alderson-Day and Fernyhough’s 2015 survey, there is a substantial body of findings on covert self talk. For example, Baddeley et al. (2001) gave their adult subjects elementary arithmetic exercises: increase or decrease a one-digit number by one. These exercises were presented either in the standard format (left) or without the usual signs (right):

$$\begin{array}{cc} 7 + 1 = {\ldots} & \qquad 7 \quad 1 \quad {\ldots} \\ 5 - 1 = {\ldots} & \qquad 5 \quad 1 \quad {\ldots} \\ 2 + 1 = {\ldots} & \qquad 2 \quad 1 \quad {\ldots} \\ 3 - 1 = {\ldots} & \qquad 3 \quad 1 \quad {\ldots} \\ \!\!\!\! {\vdots} & \quad \vdots \end{array} $$

In the standard format, this task is obviously trivial, but on the face of it at least, the signless format wouldn’t seem to make it much harder, even if it required subjects to keep mental track of the + / − alternation. Subjects had to do these exercises either with or without a concurrent verbal task, which was to recite the days of the week or the months of the year.

Baddeley et al. report that, as one might expect, subjects solved these exercises swiftly and reliably in nearly all conditions, with one exception only: with the signless format, the concurrent verbal task slowed subjects down by 60%. This is in line with the results of several studies showing that irrelevant verbalisation affects task-switching performance, while other concurrent tasks do not (e.g., Miyake et al. 2004; Saeki and Saito 2009). These findings may be explained on the assumption that irrelevant verbalisation interferes with the self talk that supports task switching. Apart from the fact that this study elegantly demonstrates how covert self talk may be detected, it also shows that a task need not be exceptionally hard to elicit self talk, even in adults.

Thus far, I’ve been using the term “self talk” dichotomously with “social talk”. It is time to set this straight: between them, social talk and self talk do not exhaust the possible uses of language. Take, for example, imagined talk:

We often imagine dialogue with aspouse, parent, sibling, or any significant other that helps us to rehearse anticipated interaction, ruminate about previous encounters, and relieve pent-up feelings that may have resulted in anxiety, conflict or tension. (Honeycutt 1995: 65)

As argued by Gregory (2016), imagined talk and self talk are quite different. In one of the scenarios Gregory uses to explain the difference (p. 664), a politician imagines herself giving a speech (“The roads in this community have been neglected for too long”), and then pauses to give herself stage directions (“I have to remember to address the audience directly”). These two modes of inner speech are clearly distinct: urging yourself to address your audience directly is not at all the same thing as imagining yourself addressing an audience. Most importantly, while in the first mode the speaker addresses herself, in the second mode she doesn’t address anybody at all; she merely imagines doing so.

For another example, rehearsing a phone number to keep it “in mind” for a brief while is a non-social activity, but on the face of it, at least, it is neither self talk nor imagined talk. Social uses of language come in many flavours, and the variety of non-social uses of language is likely to be rich, too. Hence, in all probability, self talk is just one amongst many forms of non-social talk. Given that the phenomenology of non-social talk is still in its infancy, it is anybody’s guess how frequent this particular form really is. Still, it is doubly significant that there should exist a variety of non-social talk that involves addressing an audience (i.e. oneself). First, because this is a defining feature of social talk, too, which suggests that self talk may be more closely related to social talk than other forms of non-social talk. Secondly, because this feature of self talk is a major embarrassment to the received view on communication, as we are about to see.

## Self Talk and Information Exchange

The idea that communication is information exchange is so well established that the Oxford English Dictionary gives the first meaning of “communication” as “the imparting or exchanging of information by speaking, writing, or some other medium”. On this definition, the essence of communication is that information is passed from a sender to a receiver. Basic examples are the use of colours for indicating temperatures (red for hot, blue for cold) or regulating traffic flow (red for “Stop!”, green for “Go!”). Information-based analyses of speech acts come in many flavours, but as the differences don’t matter here, we might as well pick a simple one:

1. When I say to you, “Well done!”, I thereby send you the information that you did a good job, and communication has succeeded if you receive the information that you did a good job.

When applied to self talk, this style of analysis consistently yields oddities like the following:

(2) When I say to myself, “Well done!”, I thereby send myself the information that I did a good job, and communication has succeeded if I receive the information that I did a good job.

This looks wrong, and it is evident why. Transmitting a piece of information *φ* from A to B requires that A has *φ* to begin with, and is pointless if B has *φ* already. Therefore, on an information-based analysis, telling myself that I did a good job is redundant by definition, and the same will hold for any speech act that I address to myself: if communication is information exchange, *all* self talk is redundant.

This conclusion does not depend on the kind of information that is being transmitted. To underline this point, consider a different style of analysis, still within the information-based paradigm, which adopts the widely held view that speech acts express speakers’ mental states: their beliefs, desires, intentions, and so on. Most theories that view communication as information exchange are expressivist in this sense of the word. (Harnish 2009 surveys speech act theories under this aspect, and reports only one exception.) On an expressivist account, we might have analyses like the following:

(3) When I ask you, “What’s the time?”, I thereby express that I want you to tell me the time, and communication has succeeded if you understand that I want you to tell me the time.

As expressivist analyses go, this is rather austere, as such analyses tend to recruit mental-state verbs in greater numbers. Still it has the typical expressivist format: the speech act expresses a mental state on the part of the speaker, and communication succeeds if the hearer understands that that mental state has been expressed. Extrapolating from social talk to self talk, this yields:

(4) When I ask myself, “What’s the time?”, I thereby express that I want me to tell myself the time, and communication has succeeded if I understand that I want me to tell myself the time.

This sounds at least as bad as (2), and for the same reason. In fact, the expressivist analysis may sound worse, presumably because it depicts the speaker as informing himself about his own mental state. At any rate, this case illustrates that, on an expressivist account, too, all self talk is redundant; which is ironical, since one should have expected that explaining “verbal thought”, as self talk is often called, would be a picnic for a mentalist account of communication.

So the conclusion is that self talk is a problem for the information-based theories of communication. But aren’t there any escape routes? There are a few that readily suggest themselves, but as far as I can see, there is only one that deserves to be taken seriously. Let’s briefly consider the unserious ones first. “If self talk seems to be a problem for information-based theories of communication, then that’s because self talk is a non-communicative practice. After all, communication is information exchange, and information exchange is pointless when sender and receiver coincide.” Whatever relief this argument may seem to offer is merely terminological. We are looking to explain how self talk can be continuous with social talk, in the sense that the transition from the latter to the former is natural and smooth. Being told that, unlike social talk, self talk is not for communication does not bring us closer to an explanation of that fact, and merely confirms that self talk is a problem for the received view on language use.

“Yes, self talk *is* anomalous. But that doesn’t mean the information-based paradigm is wrong. After all, reminders are anomalies, too, and for much the same reason as self talk; but it would be silly to say that reminders require us to reconsider what communication is.” Contrariwise, I don’t deny that communication implies information exchange. It would be foolish to do so. I don’t even deny that communication *invariably* implies information exchange (nor do I deny that it invariably implies elementary particles moving about). In that sense, I have no quarrel with the information-based paradigm. I’m just questioning the notion that viewing communication as information exchange is the best way of understanding what communication is and how it works. In this I’m following the lead of such luminaries as Austin, Wittgenstein, and Trump, so I’m not on my own.

Moreover, the analogy between reminders and self talk is flawed. For one thing, whereas reminders are merely one subtype of speech act, self talk is a common practice. Therefore, whereas the fact that reminders are intrinsically redundant need not worry us too much, it would be a great deal more disturbing if all self talk was redundant, which it is on any account that defines communication as information exchange.

The third and most substantial argument I will consider is that, appearances notwithstanding, self talk is a dyadic activity after all: when I talk to myself, speaker and addressee are actually distinct. This is argued as follows. It is widely agreed that the mind divides into compartments, variously called “modules”, “systems”, “selves”, “personae”, and so on. The theoretical conceptions associated with these labels are highly diverse, but they all open the door to the view that self talk is an activity *within* rather than *of* a person: whereas social talk is *inter* personal communication, self talk is *intra* personal communication.^3^

This argument amounts to a straight dismissal of what I consider to be the quintessence of self talk, which is that it involves addressing oneself. What evidence is there that self talk, thus understood, doesn’t exist? Empirical evidence strongly supports the premiss that we talk to ourselves, and although it remains unclear how common self talk is, there is no evidence I know of to show that it doesn’t exist at all. Moreover, the distinction between interpersonal and intrapersonal communication is predicated on an equivocation: communication between people is an entirely different beast from communication between mental compartments, regardless whether you call them “modules”, “systems”, or even “personae”. Therefore, to replace the notion of self talk with that of intrapersonal communication is to deny the fact that (what would appear to be) self-directed uses of language are continuous with other-directed uses of language; which is a steep price to pay.

In addition to being equivocatory, the appeal to intrapersonal communication involves a shift between levels, too. For reasons given in the introduction, my working hypothesis is that the continuity between social talk and self talk is best explained in terms of speech acts. On my view, the key issue is what might be the rationale of reproaching oneself, asking oneself questions, giving oneself orders, and so on. In contrast, by replacing self talk with intrapersonal communication, one slides from the level of linguistic action to the processing level, at which the continuity issue cannot even be coherently expressed, since mental compartments don’t perform speech acts.

In this section I have elaborated what I take to be a straightforward observation, namely, that self talk is a predicament for the information-based approach to communication. In the following, I present an alternative paradigm which doesn’t deny that speech acts carry information, but does suppose that viewing communication as information exchange is not the best way of understanding what communication is and does. What holds for the information-based approach in general, holds for its expressivist satellites, too. Again, it is undeniable that speech acts can carry information about speakers’ mental states, but that doesn’t vindicate the position that communication is bound to fail unless the addressee grasps the mental state expressed by the speaker.

As a corollary, I oppose the widely held view that, inevitably, a sincere speech act must be preceded by the mental state it expresses. It is perfectly coherent to hold that a self-addressed statement may be a way of forming a belief, that a self-addressed command may be a way of forming an intention, and so on. The idea that these notions are incoherent is an offshoot of the expressivist doctrine, and loses whatever force it may seem to have when we abandon that view, as I have argued we must. That said, in this paper I will neither defend nor presuppose that self-addressed speech acts may serve to form intentions and beliefs. However, my theory is consistent with that possibility, which I consider to be an important selling point.

## Commitments

Of necessity, social agents must coordinate their actions, not only to attain shared goals, like playing a string quartet, doing the dishes together, or making holiday plans, but also to be able to share an office, live in the same house, or drive on the same road. Commitment is a *sine qua non* for action coordination: social agents must rely on each other to act in some ways and refrain from acting in others. Commitments are coordination devices, and the main purpose of communication is to establish commitments.^4^

Although this way of looking at communication does not represent the majority view, commitment is a common enough concept in the philosophy of language, rhetoric, speech act theory, and formal theories of dialogue (see de Brabanter and Dendale^2008^ for a survey and references). As I use the term, commitments belong to the same family of relations as obligations, duties, and responsibilities, all of which are primarily towards others, but may also be towards oneself (according to some philosophers, we all owe it to ourselves to be good, for example). It is somewhat unfortunate that, in addition to this use, the English word “commitment” has a related use that is psychological, connoting dedication and resolve. That is not what I’m driving at. For me, commitments are obligations, and although they may be underwritten by suitable mental states, it is not necessary that they are. Insincere commitments are as binding as sincere ones, and there are unintended commitments, too. If I raise my hand at an auction, I thereby commit myself to be making a bid for whatever is currently under the hammer, even if I have no intention of doing so. True, I can try to get out of my commitment, for example, by arguing that I was only waving away a fly, but that presupposes there is a commitment to be undone.

Commitment is a triangular relation between two individuals and a propositional content, i.e. a possible state of affairs. The commitments we are specifically interested in are brought about by speech acts performed by a speaker and directed at an addressee.^5^ Promises are the paradigm case. If Mel promises Don to bring him his newspaper, then Mel becomes committed to Don to act so that the proposition, “Mel will bring Don his newspaper”, becomes true. Due to Mel’s committing herself in this way, Don becomes entitled to act on the premiss that he will get his newspaper, and thus Mel’s commitment helps Don to coordinate his actions with Mel’s.

Other speech act types similarly give rise to commitments on the part of the speaker, though as one might expect, the details vary from type to type. Consider statements. I say: “It snowed in April.” The content of my commitment is a possible state of affairs in the past, but still my speech act constrains my *future* actions: I’m committed to act in the future on the premiss that it snowed in April. Various kinds of obligations flow from this. Being so committed, I’m expected to be able to offer at least some justification for my statement and not to act so as to weaken it, for example, by implying that it didn’t snow in April or wondering if it did. Granted, I am allowed to change my mind and renege on my commitment, but if I choose to do so, I’m expected to be explicit about it; that, too, is part of my commitment.

Whereas the content of a promise is a goal of the speaker, and must therefore lie in the future, the commitments created by statements are not goal-directed, and may be about any possible state of affairs, past, present, or future. We’ll say that, while promises give rise to telic commitments, the commitments created by statements are atelic. The distinction is an important one, but it bears emphasising that, whether telic or atelic, commitments always act as self-imposed constraints on our future actions, thus enabling others to coordinate their actions with ours.

We undertake commitments so as to constrain our actions, though not in the rigid sense that, once we have made a commitment, we stick to it come what may. Whether I tell you that Belgium is a republic or promise to repay my debts to you, my commitment persists *ceteris paribus*, but it is renegotiable, for example, if I should discover that Belgium has a king or that I’m broke. Commitments are inert, but not immutable.^6^

## Private Commitments

To return to our main topic, we are casting about for a unified analysis of other- and self-directed utterances that should allow us to model speech acts of the latter category as deriving from speech acts of the former, the main difference being that the speaker addresses herself rather than another. The theory outlined in the foregoing makes straightforward predictions about the effects of self-directed speech acts: they will give rise to *private* commitments of the form, “The speaker is committed to herself to…” Like all commitments, these will be either telic or atelic. To show how private commitments account for the self-directed uses of speech acts, we first consider promises. By promising Mel, “I’ll mow the lawn today”, Don commits himself to Mel to mow the lawn today. Analogously, by promising himself, “I’ll mow the lawn today”, Don makes a commitment to himself to mow the lawn today, and his commitment is private and telic; that is to say, it is a commitment to himself with a goal state that only himself can bring about (only Don can make it happen that Don mows the lawn).

What is the point of a private commitment? It’s the same as in the social case: commitments enable coordination. Don’s promise to Mel entitles her to plan her activities on the assumption that Don will mow the lawn today, so she can take it as given that she will not have to do it herself, that Don will be out of the house for at least an hour, and so on. Likewise, Don’s promise to himself entitles him to make his plans on the assumption that he will mow the lawn today, so he will not have to ask Mel to do it, he will be busy for at least an hour, and so on. In short, while Don’s commitment to Mel enables Don and Mel to coordinate their respective actions, Don’s commitment to himself enables him to coordinate his own actions.

Though it may seem tempting to view Don’s private commitment as a compact between his present self and some future self, that temptation is better resisted. Suppose at time *t* Don undertakes a commitment to Mel to mow the lawn at *t* ^′^. The propositional content of Don’s commitment is that he will mow the lawn at *t* ^′^, but it is evidently not a relation between Don-at-*t* and Mel-at-*t* ^′^. Rather, it is a relation which starts to hold at *t* and persist until it is either discharged or cancelled. The same holds for Don’s commitment to himself.

More likely than not, private commitments will tend to be less robust than social ones, for the simple reason that maker and recipient coincide, and in general it is much easier to bargain with oneself than with others. Still, this does not render private commitments useless, as they remain indispensable for planning one’s activities. While it may be true that one can afford to renege on practically any commitment to himself, a large-scale cancellation of private commitments is guaranteed to cause planning paralysis; it just can’t be done. Commitments to others may enjoy more stability than commitments to oneself, but the difference is merely one of degree.

The case of Don’s self-directed promise illustrates how a commitment-based approach to speech acts allows us to make sense of self talk. Let us now take this little case study to its conclusion. Our objective is to explain how speech acts, those epitomes of social interaction, can be directed to oneself and serve as psychological acts. How is it possible for Don to take what is originally a social act, “I’ll mow the lawn today”, direct it to himself, and thereby form the intention to mow the lawn?^7^ The answer is simple: by making a promise to oneself, one privately commits to a goal, and telic private commitments *are* intentions. This claim is motivated by two considerations. One is that it agrees with our pre-theoretic intuitions: as a matter of linguistic fact, it seems correct to say that if I privately commit to stop drinking, for example, then I intend to stop drinking. Secondly, the concept of telic private commitment practically coincides with Bratman’s (1987) concept of intention; which must count as an argument, because Bratman’s analysis is widely accepted as the gold standard amongst theories of intention.

On Bratman’s account, intentions are like desires in that they motivate us to act, but unlike desires, intentions imply resolution. I may want to have a drink, yet resist the temptation, but once I have resolved to get a drink, I have an intention; I have “made up my mind”.^8^ A key trait of intentions, according to Bratman, is their inertia. If I intend to take my bike in for service at noon, then of course I can still change my mind, but by default I will stick to my intention. The ability to settle on a course of action in advance, albeit defeasibly, has at least two advantages. First, by settling on a course of action beforehand, my intentions allow me to make deliberate decisions under circumstances that leave no time for deliberation, simply by doing my thinking in advance. Secondly, my intentions enable me to coordinate my activities: having formed the intention to take my bike in for service at noon, I plan my activities for the day accordingly, treating it as given that I will take my bike in for service at noon.

All this chimes beautifully with what I have been saying about telic private commitments. The main difference between my account and Bratman’s is that mine is more general. Abstracting away from the differences between coordinating one’s own actions and coordinating one’s actions with others’, it treats telic private commitments (Bratman’s intentions) as merely special cases of a more general category, which further subsumes atelic private commitments as well as social commitments. An intention à la Bratman is a commitment to oneself to achieve a certain goal.

Turning to private commitments of the atelic variety, consider the following scenario. Mel is trying to remember where her phone is. She weighs and rejects various possibilities, until she finds herself left with one option only, and concludes: “It must be in the kitchen.” By telling herself that her phone must be in the kitchen, Mel addresses a statement to herself, thus forming a atelic private commitment to act in accordance with the premiss that her phone is in the kitchen.^9^

Atelic private commitments are useful in much the same way that their telic counterparts are. Both are part of the relatively stable background against which we coordinate our own actions. Once Mel has told herself that her phone must be in the kitchen, she will persist, if only by default, to act in accordance with the assumption that her phone is in the kitchen. Thus, if she needs her phone, she will go into kitchen to fetch it; when asked where her phone is, she will say it is in the kitchen; if the kitchen is on fire, she is likely to infer that her phone is in jeopardy; and so on. In short, by telling herself that her phone is in the kitchen, Mel has formed the belief that it is so. For, if you are privately committed to the truth of a proposition, then you believe that it is true: atelic private commitments are beliefs.

Although there are a wide variety of doctrines on the nature of beliefs, the claim that atelic private commitments are beliefs does not commit us to any particular one of them. The proper function of commitments, be they telic or atelic, is to constrain their owners’ action potentials. To say that atelic private commitments are beliefs is to imply that beliefs tend to constrain their owners to activities, external as well as internal, that respect the truth of a given proposition. It seems to me that this much is uncontroversial and compatible with just about any theory of belief, excepting only those which deny that beliefs exist in the first place.

The foregoing discussion highlights two important parallels between intentions and beliefs. It is generally agreed that intentions and beliefs guide our actions and interactions: we act on our beliefs and intentions. What seems to be less widely realised is that in order to fulfil that purpose, beliefs must be inert, just as intentions must be. The famous false-belief task illustrates this (Wimmer and Perner 1983). In the canonical version of this task, Sally puts a marble in her basket and goes out for a walk. While she is away, Anne takes the marble out of the basket and puts it in her box. Then Sally reappears, and subjects are asked the critical question: “Where will Sally look for her marble?” It is well established that young children tend to fail this task: rather than pointing to Sally’s basket, they point to Anne’s box. The normative response, that subjects should point to the basket, is motivated by reasoning that that is where Sally last saw the marble, and since she wasn’t given any reason to change her opinion, she must still believe that it is there. Hence, it is presupposed that Sally’s belief is inert. It is their inertia that enables us to use beliefs and intentions as coordination devices.

Thus far, we have confined our attention to promises and statements. In the remainder of this section, we extend our scope to directives, a category of speech acts that includes orders and requests, but also questions, which will be our focus of interest. Since questions will be treated as a special type of directive, we start with an order. If Mel tells Don, “Walk the dog!”, her goal is that Don walk the dog, and Mel commits herself to that goal, which entails that, to the extent that Don’s walking the dog depends on Mel’s cooperation, he is entitled to expect her to help. If Don cannot find the dog, for example, and Mel knows where it is, then Don may expect her to tell him. Hence, directives resemble promises in that they create telic commitments on the part of the speaker. They differ from promises in that their goal states can only be made true by the addressee: when directed to Don, the goal state of, “Walk the dog!”, is that Don walks the dog, which is a proposition that only Don can make true.

It follows from this analysis that, when addressed to oneself, promises and directives create the same commitments; for, when speaker and addressee coincide, only the speaker can make the goal state true in either case, thus forming the intention to bring about a certain state of affairs. Hence, we predict that, when addressing himself, Don will create the same commitment for himself by ordering or promising himself to walk the dog. This seems to be correct.

Turning to questions, if Mel asks Don, “Are you gay?”, she doesn’t merely expect him to say either yes or no: she expects him to commit himself to the truth of either the proposition that he is gay or its denial. More precisely, Mel’s question causes her to become committed to the goal that Don commit himself either to being gay or to not being gay (Krifka 2015). If Don is prepared to commit himself on this issue, he may do so simply by saying yes or no, though there are other ways, too.

This analysis predicts that by asking himself, “Am I gay?”, Don becomes committed to the goal of committing himself either to being gay or to not being gay. This would be a useful thing for Don to do, for example, if he had been avoiding the issue for some time, and then decided that he must finally make up his mind. When employed in this way, self-addressed questions serve to coordinate reasoning processes, and they do so in two respects. First, they help to direct a train of reasoning by establishing issues to be addressed. For example, in order to decide for himself whether he is gay, there are various subquestions that Don may choose to deal with, any of which may give rise to subquestions of its own, and so on. Secondly, in their ordinary, other-directed use, questions are typically answered immediately, and it seems plausible that this is what speakers have come to expect by default. Assuming that this expectation carries over to self talk, if someone asks himself a question, he thereby becomes committed to answer it without undue delay. Hence, a self-addressed question not only puts an issue on one’s mental agenda, it also urges that the issue be dealt with shortly; this is one of the ways in which self talk is used to focus one’s attention (cf. Fernández Castro 2016). Thus the commitment-based approach to communication enables us to flesh out the idea that some of modes of thinking are dialogical processes of asking questions and giving answers, an idea that we find already in Plato.

## Conclusion

The main objective of this paper was to explain the continuity between social talk and self talk by means of a theory that equally covers other-directed and self-directed speech acts. The proposed account delivers such a unified analysis. Its fulcrum is a concept of commitment that straddles two dichotomies: telic/atelic and private/social. On the one hand, a commitment is a self-imposed constraint on an individual’s future actions, whose satisfaction may but need not be amongst his goals. In the former case, the commitment is telic; in the latter, it is atelic. In both cases, commitments are made in order to coordinate actions, either of an individual or between individuals. On the other hand, commitment holds between pairs of individuals that may or may not coincide. In the former case, the commitment is private; in the latter, it is social.

Both aspects are critical to my enterprise. On the one hand, due to fact that it covers both telic and atelic instances, commitment may serve as a foundational element in a theory of communication. Such a theory is expounded at some length in Geurts (2017a), and was illustrated here by applying it to four kinds of speech acts representing the three major speech act types: promises (commissives), statements (constatives), and questions and orders (directives). On the other hand, due to fact that commitment covers both individual and social instances, we can explain the continuity between self talk and social talk. Communication starts out as a social practice, a way of making commitments to others, but on the proposed account it is straightforward as well as sensible to turn this practice around and start making commitments to oneself. Thus it is explained why self talk comes naturally to us and why it makes sense.

## Footnotes

1 See Geurts and Rubio-Fernández (2015) for discussion of the relation between pragmatic theories and processing models.

2 Much of the previous research on self talk is focused on inner speech. To my knowledge, there is no evidence that the dichotomy between inner and outer speech is of any great significance, and therefore I prefer the term “self talk”, which is neutral between “inner” and “outer”.

3 Ruesch and Bateson (1951) were early adopters of this view. Vicente and Martinez Manrique (2011) discuss several of the more recent accounts.

4 Due to space limitations, the following outline of a commitment-based theory of communication is just that: an outline. See Geurts (2017a) for a full-dress discussion, which elaborates on the notion of commitment and the analysis of speech acts, develops a theory of common ground in terms of mutual commitments, and shows how to accommodate a Gricean treatment of cooperativity and conversational implicatures in a commitment-based framework. Geurts (2017c) discusses fiction and pretence in terms of commitments.

5 In this paper, I will not be concerned with the question how sentence tokens come to be associated with speech acts. Considerable quantities of ink have been spilled on that issue, but by now there seems to be a broad consensus that both conventions and contextual factors are involved. Elsewhere, I have argued at length that common ground is critical for the proper functioning of social conventions in general, and language conventions in particular, and on that basis developed an account of language conventions from which speech act conventions fall out as a special case (see Geurts 2015, 2017b).

6 Although in this paper I’m not concerned with the mental processes and representations that underlie communication, I should like to note that it is perfectly possible to make and act upon commitments without having the concept of commitment. By contrast, it is widely accepted that, on an expressivist account, it is a prerequisite for communication that speakers and hearers grasp the concepts of belief, intention, and so on; which raises the question how young children manage to communicate as well as they seem to do (cf. Thompson^2014^ and Moore^2017^).

7 This formulation suggests that, as a result of promising himself to mow the lawn, Don comes to have an intention he didn’t have before, viz. that he will mow the lawn. Off the record, I believe this might be the case, though it surely doesn’t have to be so: Don’s promising himself to mow the lawn might be a way of renewing or reaffirming an intention predating his speech act. On the record, though my account of self talk is consistent with this possibility, it doesn’t entail that self talk actually serves to form mental states.

8 Bratman speaks of “commitment” rather than “resolution”, but I have relabelled his concept so as to keep it separate from mine.

9 The caveat of fn. 7 applies in this case, too.
